# Human identification by medical findings in a forensic anthropology context

**DOI:** 10.1093/fsr/owae041

**Published:** 2024-08-23

**Authors:** Yara Vieira Lemos, Alexandre Neves Furtado, Adriana Zatti Lima, Alexander Santos Dionísio, Ricardo Moreira Araújo, Eugénia Cunha

**Affiliations:** Serviço de Antropologia Forense, Instituto Médico Legal Dr. André Roquette, Belo Horizonte, Minas Gerais, Brazil; Faculdade Ciências Médicas de Minas Gerais, Belo Horizonte, Brazil; Serviço de Antropologia Forense, Instituto Médico Legal Dr. André Roquette, Belo Horizonte, Minas Gerais, Brazil; Serviço de Antropologia Forense, Instituto Médico Legal Dr. André Roquette, Belo Horizonte, Minas Gerais, Brazil; Serviço de Antropologia Forense, Instituto Médico Legal Dr. André Roquette, Belo Horizonte, Minas Gerais, Brazil; Serviço de Antropologia Forense, Instituto Médico Legal Dr. André Roquette, Belo Horizonte, Minas Gerais, Brazil; Faculdade Ciências Médicas de Minas Gerais, Belo Horizonte, Brazil; University of Coimbra Centre for Functional Ecology, Laboratory of Forensic Anthropology, Department of Life Sciences; Coimbra and National Institute of Legal Medicine and Forensic Sciences, Lisbon, Portugal

**Keywords:** human identification, forensic anthropology, medical findings, legal medicine

## Abstract

This article presents a series of three complex forensic cases that posed significant challenges for identifying human remains. These include a mass dam disaster, burnt human remains, and extensively decomposed human remains. Positive identification was achieved using a shadow positioning technique with imaging comparisons of medical findings. After establishing the biological profile, medical data were evaluated with digital radiography and computed tomography examinations the human remains. These aimed to replicate the original (intravitam) traits in the same angulation to examine postsurgical characteristics, as well as the anatomical, pathological, and morphological features, which were sufficient to establish a positive scientific identification. Technological advancements tend to reveal additional skeletal details, making medical data comparisons significantly more effective in the context of anthropological identification. These cases demonstrate that the possibility of identification should never be ignored, even in situations with advanced decomposition.

**Key points:**

- Conventional identification methods may not always be applicable in forensic anthropology cases.
- The presented cases include a mass dam disaster, burnt human remains, and extensively decomposed human remains.
- These three cases involved successful human identification with medical findings comparisons using the shadow position technique.
- Identification could be established in these cases, despite challenges, such as fire damage, an incomplete body, and extensive decomposition.
- These cases suggest medical findings should be considered as biological identifiers rather than secondary identifiers.

## Introduction

Scientific identification of human remains is fundamental for understanding the perpetrator of a crime as well as for humanitarian reasons. Subsequent investigative procedures, as the assumption of motivations for a certain crime, are greatly facilitated after the victim’s name is revealed. Despite being a priority, victim identification does not occur in many forensic cases [1–3]. Numerous variables and factors can impact the decomposition rate of human remains. These include natural disasters, man-made mass disasters, and unusual and unprecedented violence, which require forensic sciences to adapt and update their research procedures to provide appropriate solutions. Despite these challenges, a holistic approach may be an effective solution to identify a body part in adverse conditions [4].

The Instituto Médico Legal Dr. André Roquette (IMLAR) forensic anthropology team (SAF/IMLAR) at Minas Gerais has been working with truly challenging cases in terms of victim identification and determining the cause and manner of death. One such case is in the Brumadinho disaster victim identification (DVI), one of the largest mass disasters in Brazil, where a catastrophic dam structural failure claimed 270 fatal victims. Because of the high rate of body segmentation, human medico-legal identification was performed concomitantly with other forensic methods that were considered to be the gold standard. In the context of Brumadinho DVI, the simultaneous identification of the same case by different methods could, in theory, test the potential of identification by medical findings [5].

Therefore, the aim of this article is to present a series of cases in which the SAF/IMLAR multidisciplinary forensic anthropology team positively identified human remains under challenging forensic circumstances. The cases include a mass dam disaster, burnt human remains, and extensively decomposed human remains.

**Figure 1:**
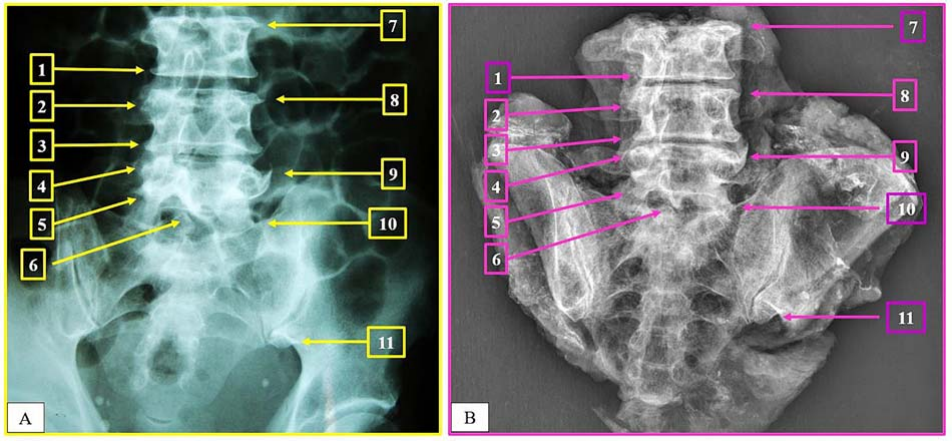
Intravitam (A) and postmortem (B) X-ray confrontation of the lumbar spine. Matching points 1–11.

**Figure 2:**
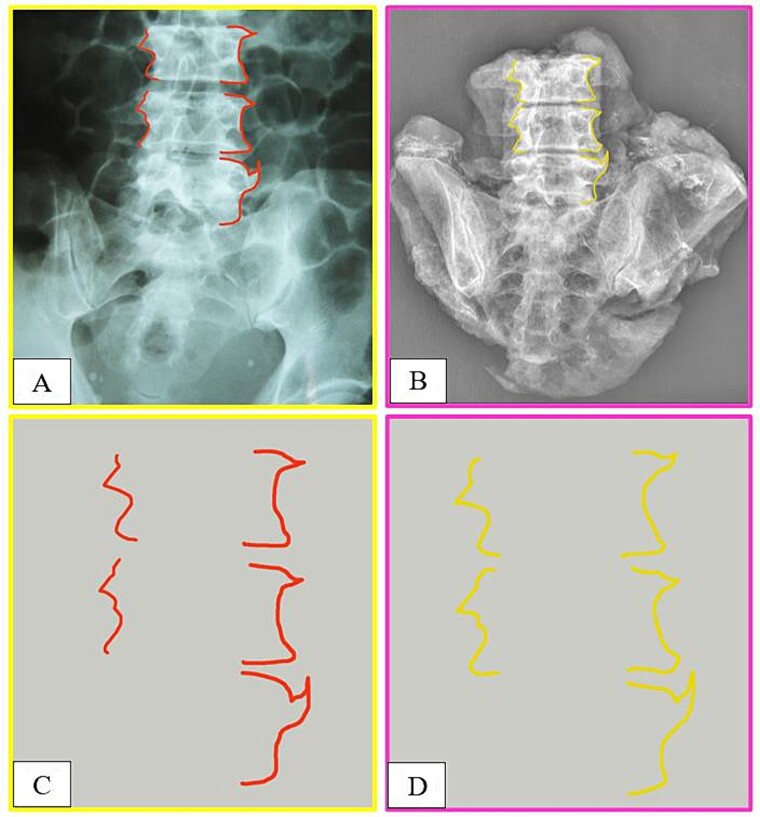
Intravitam (A) and postmortem (B) X-ray confrontation of the lumbar spine. Comparison of the antemortem (C) and postmortem (D) templates.

**Figure 3:**
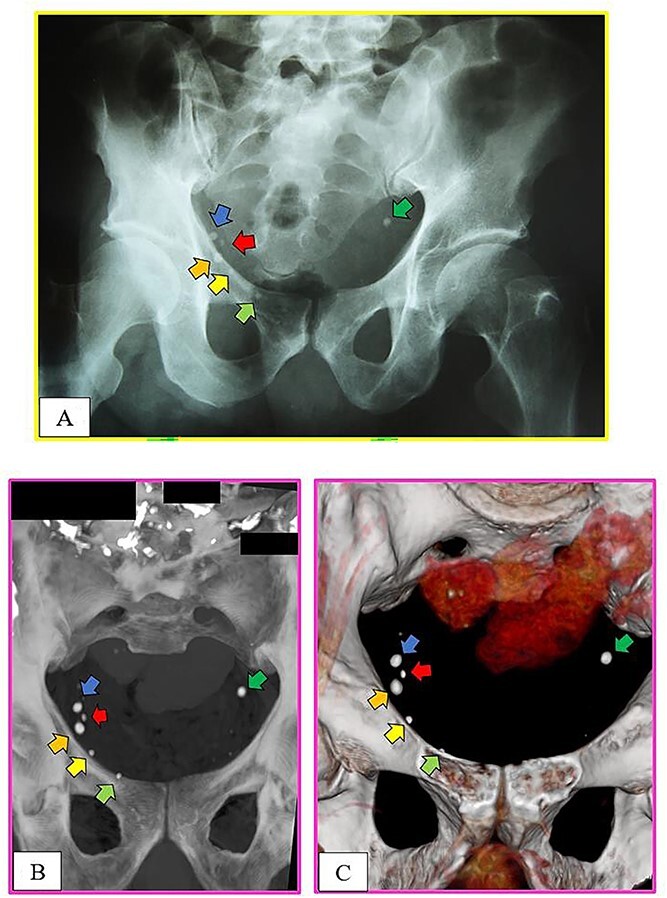
Comparisons of the X-ray and computed tomography (CT) images. Pelvic calcifications. Intravitam X-ray (A), conventional postmortem CT (B), and postmortem CT with 3D reconstruction (C).

**Figure 4:**
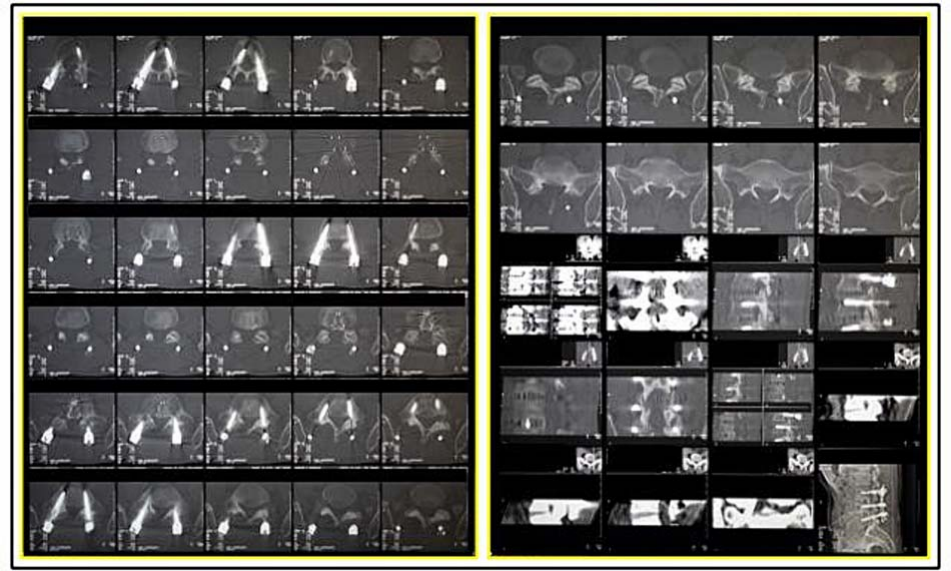
Printed tomographic study of the intravitam lumbosacral spine referred to the Instituto Médico Legal Dr André Roquette forensic anthropology team (SAF/IMLAR).

**Figure 5:**
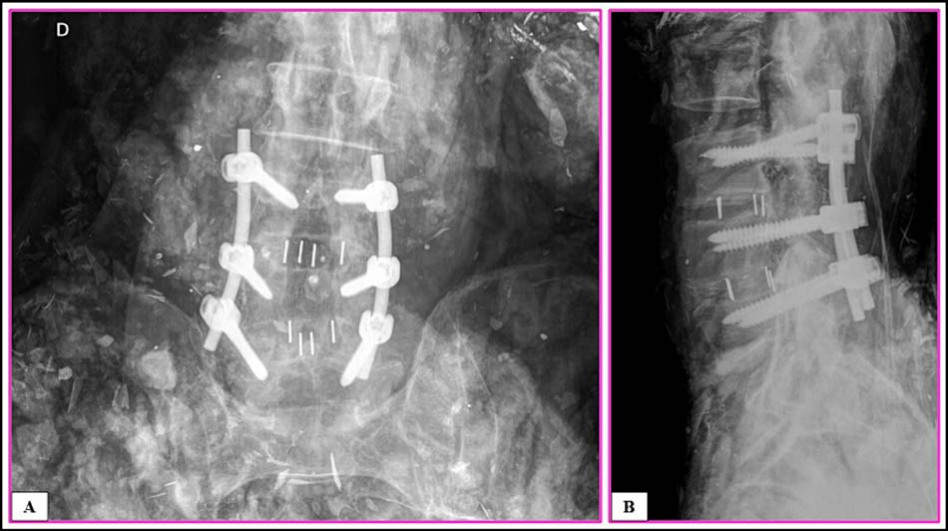
Postmortem X-ray of the lumbosacral spine in the anteroposterior (AP) (A) and lateral (B) views.

**Figure 6:**
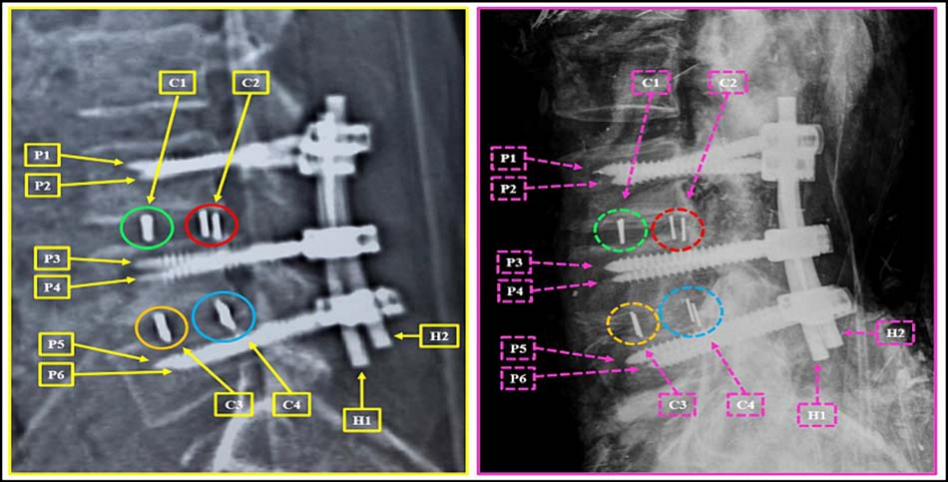
Comparison between the antemortem CT images (left) and postmortem lateral X- ray (right). Elements of agreement.

**Figure 7:**
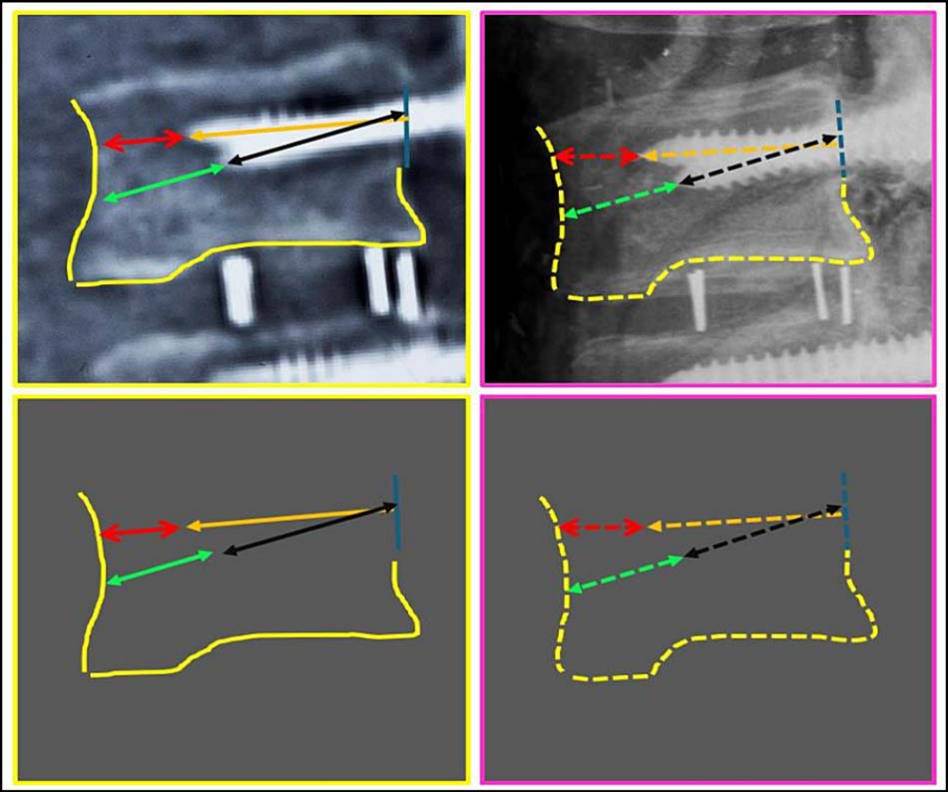
L3 vertebral body. Upper panels, comparison between the antemortem CT images (left) and postmortem lateral X-ray (right). Lower panels, respective concordant templates.

**Figure 8:**
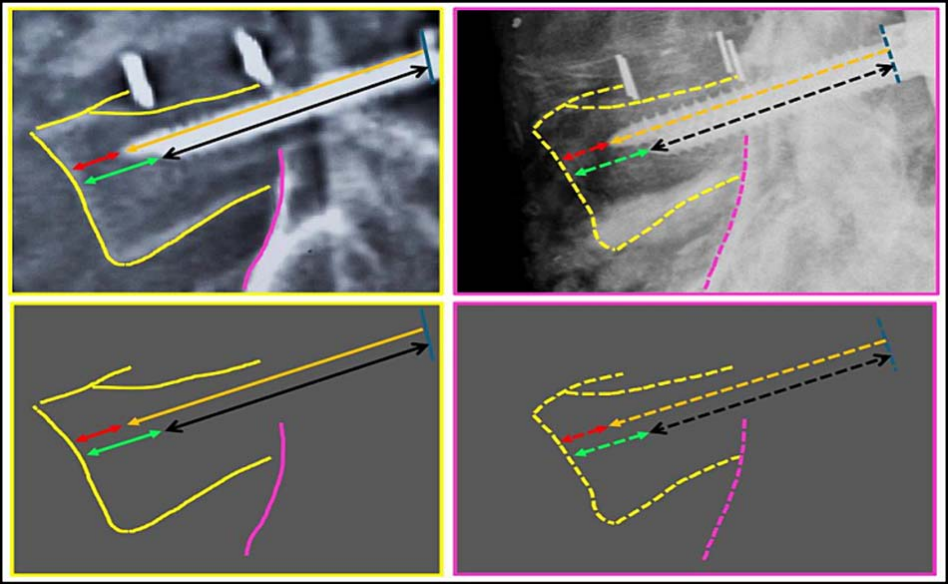
Contour of the L5 vertebral body. Upper panels, comparison between the antemortem CT images (left) and postmortem lateral X-ray (right). Lower panels, respective concordant templates.

## Case study

### Case 1

An incomplete, putrefied, partially saponified, unknown human corpse was recovered during the Brumadinho DVI case, which involved hundreds of victims. The skull and jaw were not recovered. Although 95.9% of victims of the Brumadinho mass disaster were identified within 1 year, others had not been located and remained unidentified [5]. A list of missing persons was quickly drawn up using antemortem medical, dental, and radiological files digitized and categorized by the DVI Brumadinho task force. In compliance with the IMLAR standard procedures, all human remains were previously analysed by imaging. The following case was among the individuals identified during the first year following the tragedy.

The victim’s sex was determined to be male by visualization of the remaining external genitalia. The age interval was estimated as 45.6 ± 10.4 years with phase V of the Suchey–Brooks method and as 43.0–55.0 years with phase VI of the Işcan–Loth method [6–8] after minimum preparation and direct examination of the pubic bone and anterior segment of the fourth rib. The height was established as 175.0 ± 6.9 cm after evaluating the physiological length of the femur by the Mendonça method [9]. After the biological profile assessment, the radiological examination and computed tomography (CT) scan provided further identification factors, namely osteopathological lesions at the lumbar vertebrae. The biological profile and identification factors were consistent with one of the missing persons, whose intravitam*/*antemortem file included a radiographic image in anteroposterior (AP) incidence and magnetic resonance imaging (MRI). An MRI scan could not be performed on the corpse because of a lack of availability at the medical-legal facility.

The anthropological and imaging comparative examination revealed the following multiple matching points (Figures 1–3), with no excluding points:

(1) the number, location, and morphology of osteophytes in the vertebral bodies from L3 to L5 (points 1–11) in X-ray *versus* X-ray confrontation (Figure 1),
(2) the contour of the vertebral bodies from L3 to L5 in X-ray *versus* X-ray confrontation (Figure 2), and
(3) the number, location, and morphology of pelvic calcifications in X-ray *versus* CT (Figure 3).

### Case 2

A traffic accident on a federal highway resulted in a fatally charred victim, whose intravitam CT scan showed neurological/orthopaedical surgery on L3–L5 vertebrae (Figure 4). The examination of the remains was carried out. The sex was determined to be male by visualization of the external genitalia. The age interval was estimated as 45.6 ± 10.4 years exclusively using phase V of the Suchey–Brooks method [6], as other anatomical areas normally used for age estimation were missing. A CT scan indicated that the body showed osteophytosis in the vertebral bodies. Height estimation was not possible because of the absence/destruction of the long bones and metatarsals. The radiological examination (Figure 5) and CT scan of the remains showed signs of neurological/orthopaedical surgery, namely posterior arthrodesis involving the L3–L5 vertebrae. This consisted of two longitudinal nails in the same position (H1 and H2) and two pairs of transpedicular screws with the same conformation in each vertebra. Further examination of the nails did not reveal any serial numbers.

The investigations confirmed consistency between the medical documentation of the car’s owner and the biological profile, in addition to the identification factors available in a printed tomographic study of the lumbosacral spine (Figure 6).

A postmortem replication of the imaging examinations was performed with X-rays in AP and lateral views, while a CT scan of the body’s lumbosacral spine was performed to reproduce the same position and angulation, which revealed multiple matching points (Figures 5–25).

**Figure 9:**
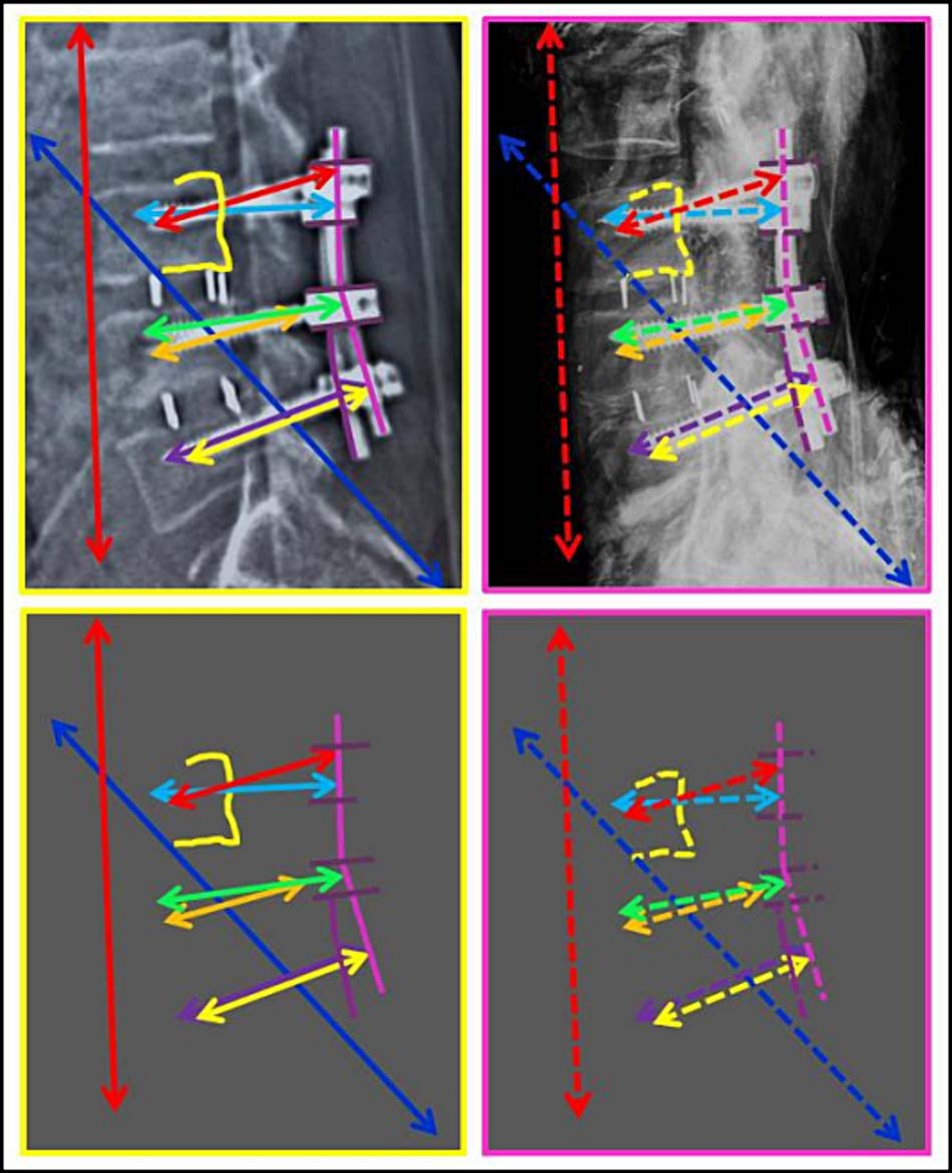
Proportion and positioning of the interposition of lines interconnecting specific anatomical points about the rods and screws. Upper panels, comparison between the antemortem CT images (left) and postmortem lateral X-ray (right). Lower panel, respective concordant templates.

**Figure 10:**
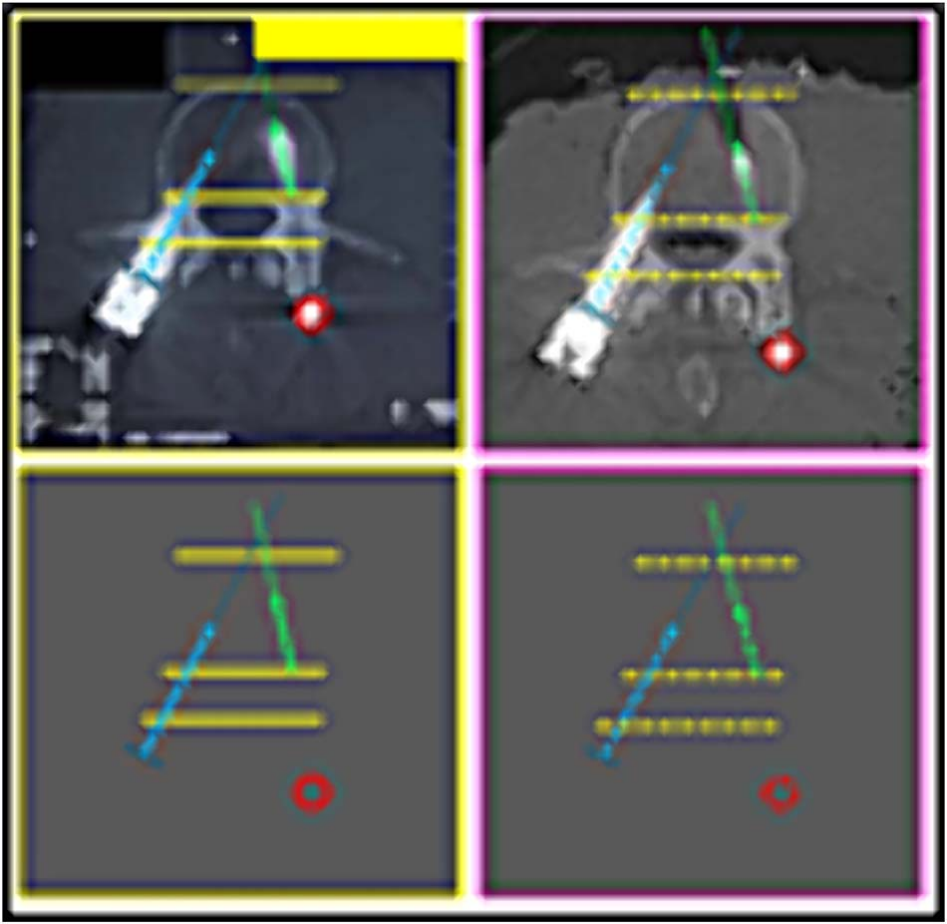
Contour of the L3 vertebral body. Upper panels, comparison between the axial slices of the antemortem (left) and postmortem (right) CT scans. Lower panels, respective concordant templates.

**Figure 11:**
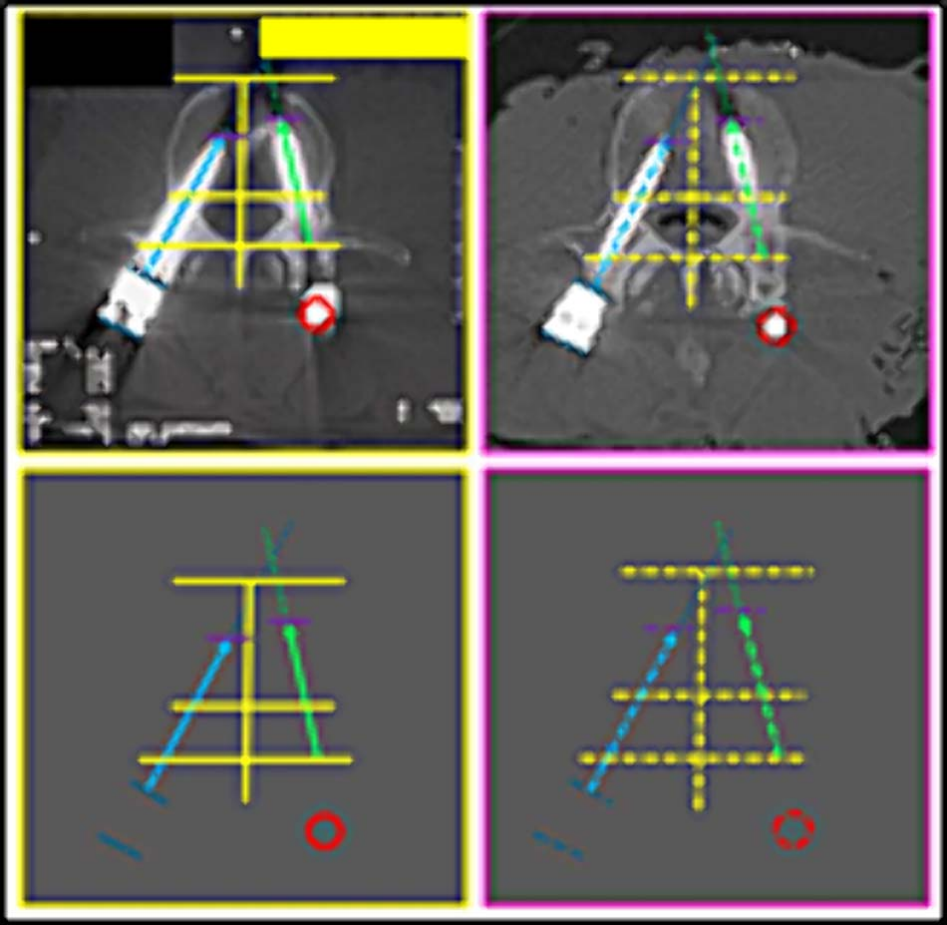
L3 vertebra. Upper panels, comparison between the axial slices of the antemortem (left) and postmortem (right) CT scans. Lower panels, respective concordant templates.

**Figure 12:**
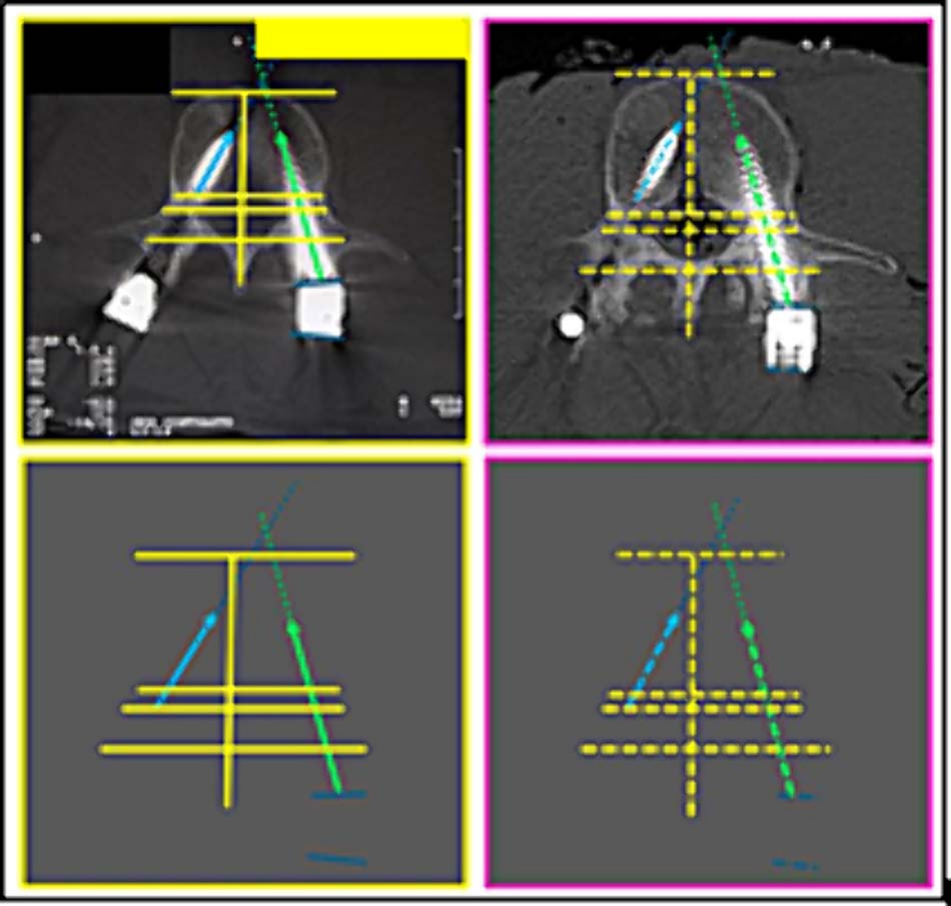
L3 vertebra. Upper panels, comparison between the axial slices of the antemortem (left) and postmortem (right) CT scans. Lower panels, respective concordant templates.

**Figure 13:**
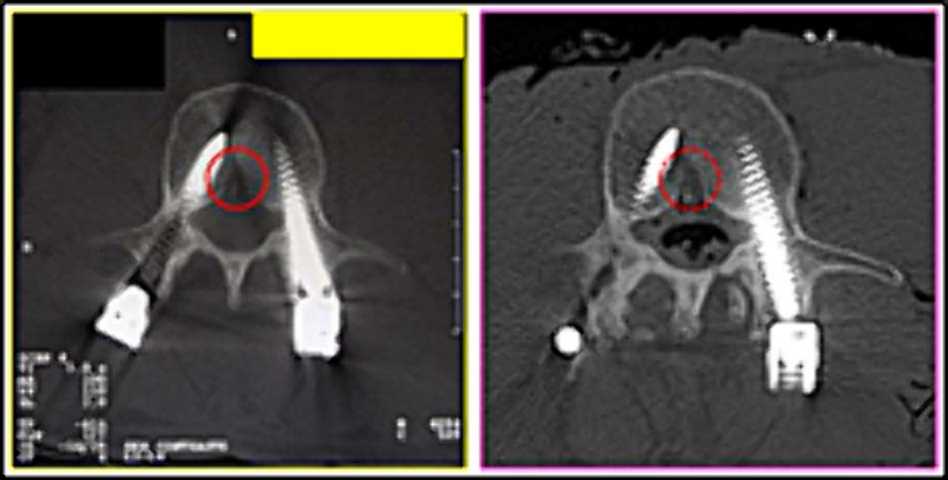
Posterior contour of the L3 vertebral body. Comparison between the axial slices of the antemortem (left) and postmortem (right) CT scans. Element of agreement.

**Figure 14:**
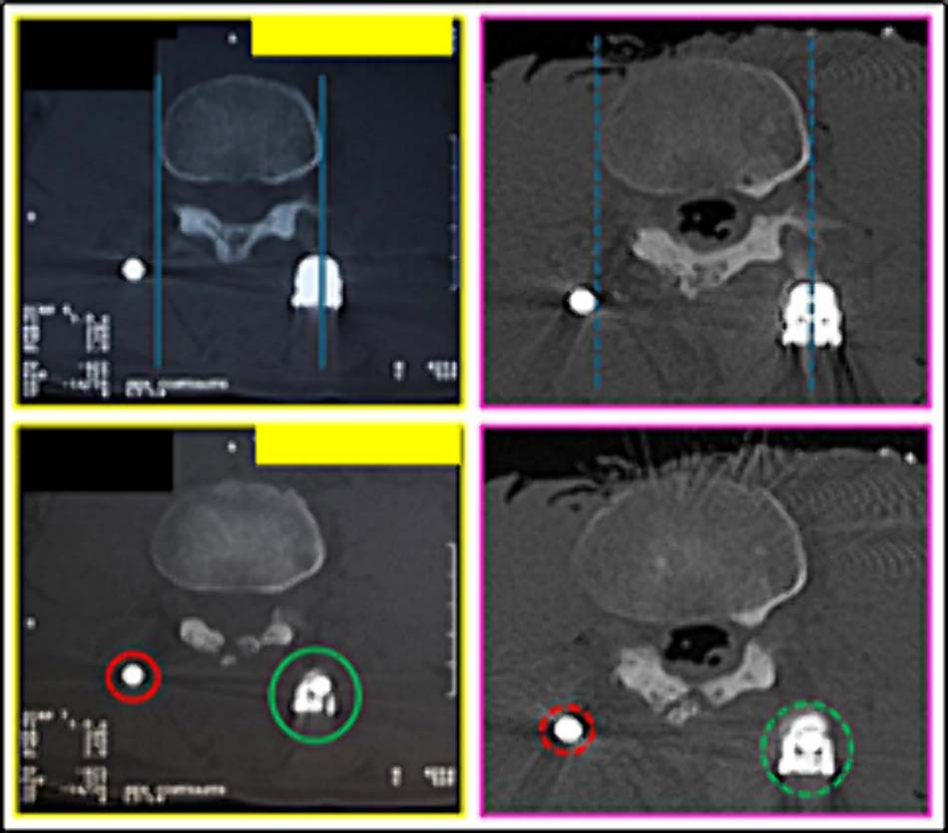
L3 vertebral body. Comparison between the axial slice images of the antemortem (left) and postmortem (right) CT scans. Consistent elements.

The anthropological and imaging comparative examination showed the following matching points, without excluding elements:

(1) posterior arthrodesis involving the L3–L5 vertebrae; these consisted of two longitudinal nails in the exact same position (H1 and H2), two pairs of transpedicular screws with the same conformation in each vertebra (P1–P6), and two pairs of disc-replacement interbody devices (“cages”) in the exact same intervertebral spaces between L3 and L4 and between L4 and L5 (C1–C4) (Figures 4–6),

(2) the contour of the L3 vertebral body; the relationship between the screws and the anterior contour of the L3 vertebral body (arrows); the angulation of the screws, individually and in groups (arrows) (Figure 7),

(3) the contour of the L5 vertebral body (upper contour) and the anterior contour of the sacrum (lower contour); the relationship between the screws and the anterior contour of the L5 vertebral body (arrows); the angulation of the screws, individually and in groups (arrows) (Figure 8),

(4) the proportion and positioning of the interposition of lines interconnecting specific anatomical points about the rods and screws (Figure 9),

(5) the contour of the L3 vertebral body (horizontal lines); the relationship of the screws with the anterior contour of the L3 vertebral body (oblique lines); the angulation of screws, individually and in groups (oblique arrows); the arrangement of one of the stems (circle) (Figure 10),

(6) the image resulting from the interposition of lines interconnecting specific anatomical points of the L3 vertebra about the rods and screws (Figures 11 and 12),

(7) the bone indentation in the posterior contour of the L3 vertebral body in the median plane of the vertebral canal (circle) (Figure 13),

(8) the contour of the L3 vertebral body (lines); the relationship of the stems (circles) with the posterior arch (Figure 14),

**Figure 15:**
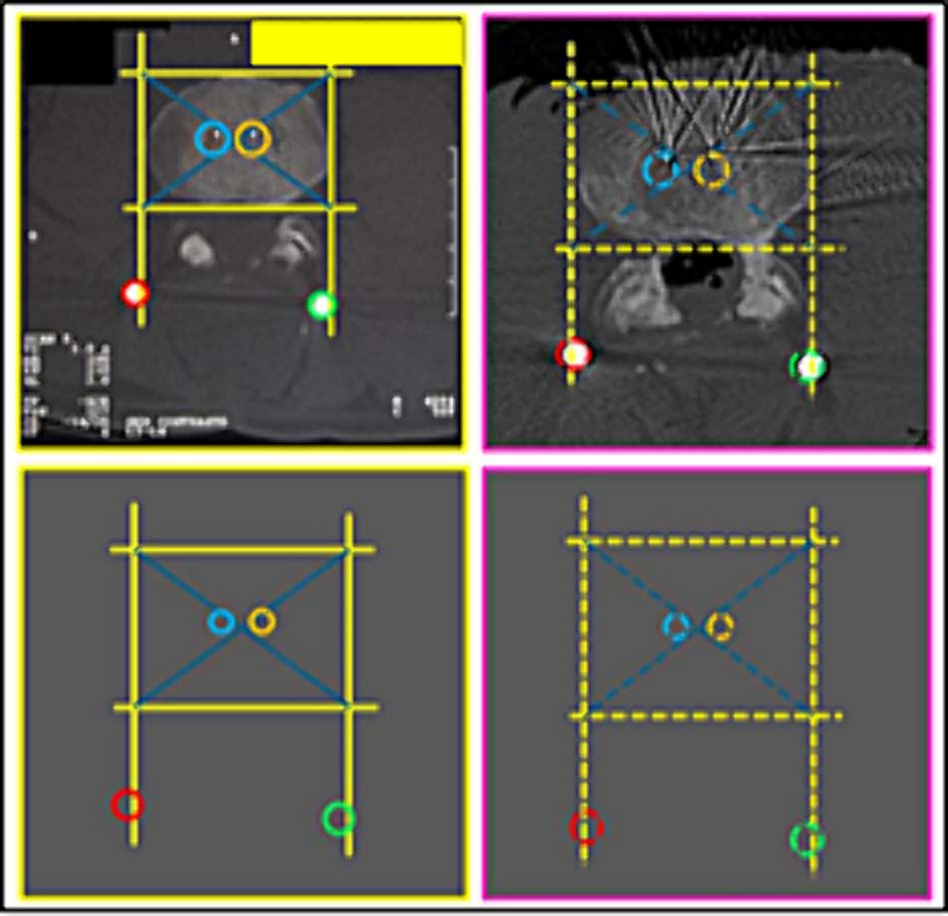
L3 vertebral body. Upper panels, comparison between the axial slices of the antemortem (left) and postmortem (right) CT scans. Lower panels, respective concordant templates.

**Figure 16:**
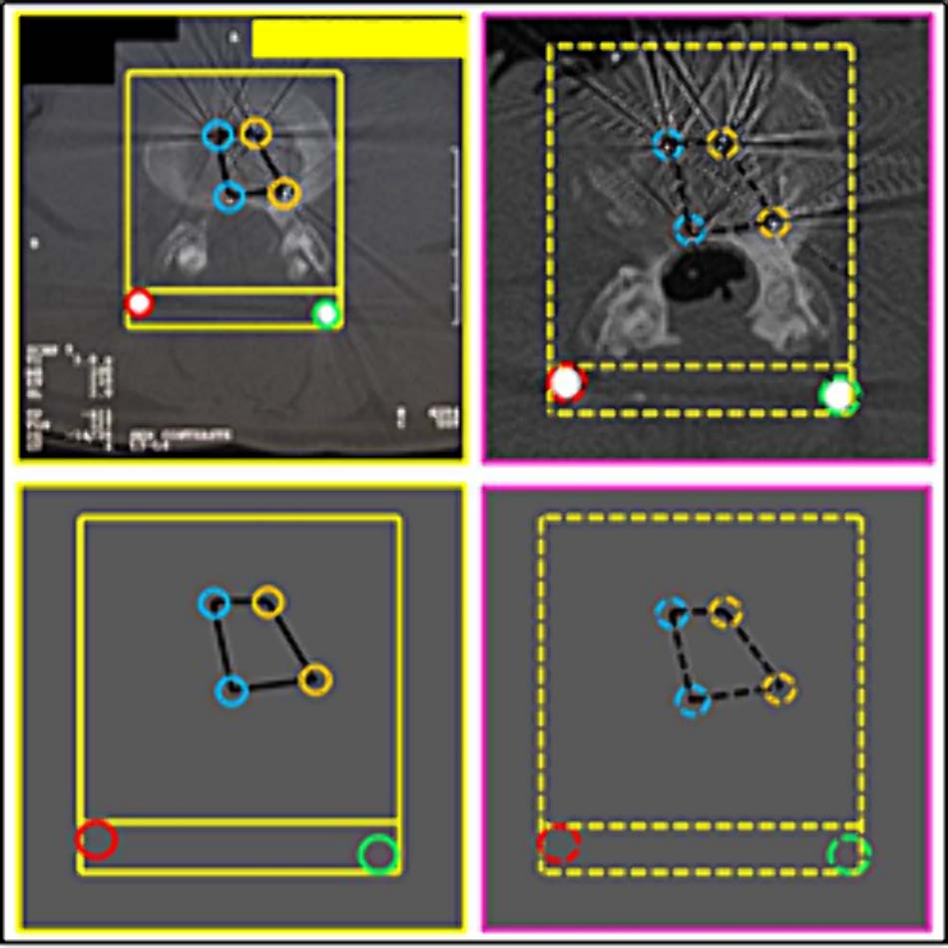
L3 vertebral body. Upper panels, comparison between the axial slices of the antemortem (left) and postmortem (right) CT scans. Lower panels, respective concordant templates.

**Figure 17:**
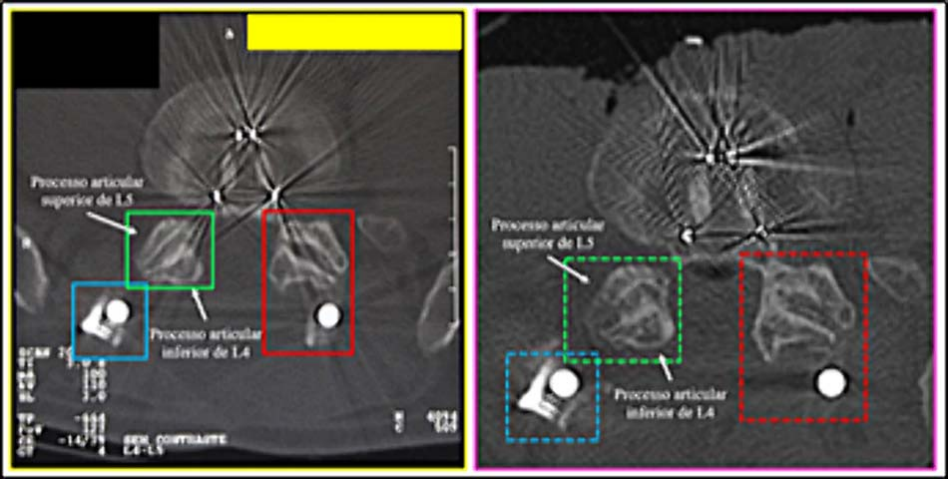
Morphology of the lower articular process of the L4 vertebra and the upper articular of the L5 vertebra. Comparison between the axial slice images of the antemortem (left) and postmortem (right) CT scans. Consistent elements.

**Figure 18:**
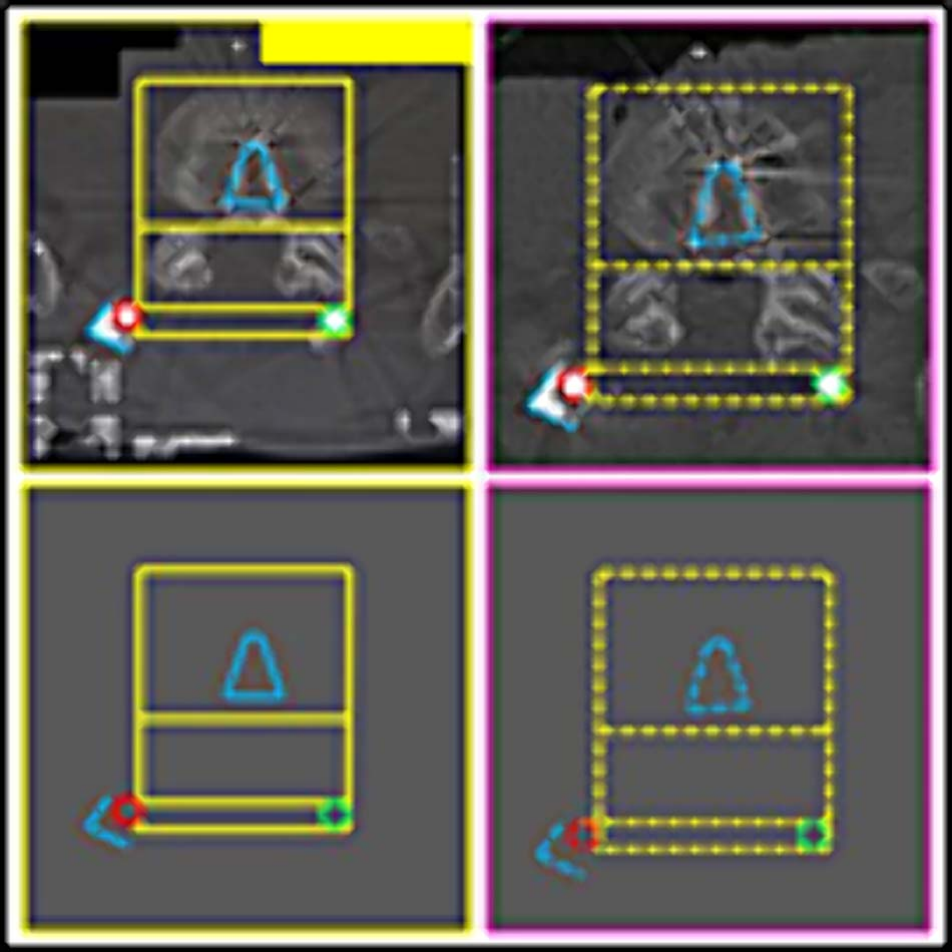
L5 vertebral body. Upper panels, comparison between the axial slice images of the antemortem (left) and postmortem (right) CT scans. Lower panels, respective concordant templates.

**Figure 19:**
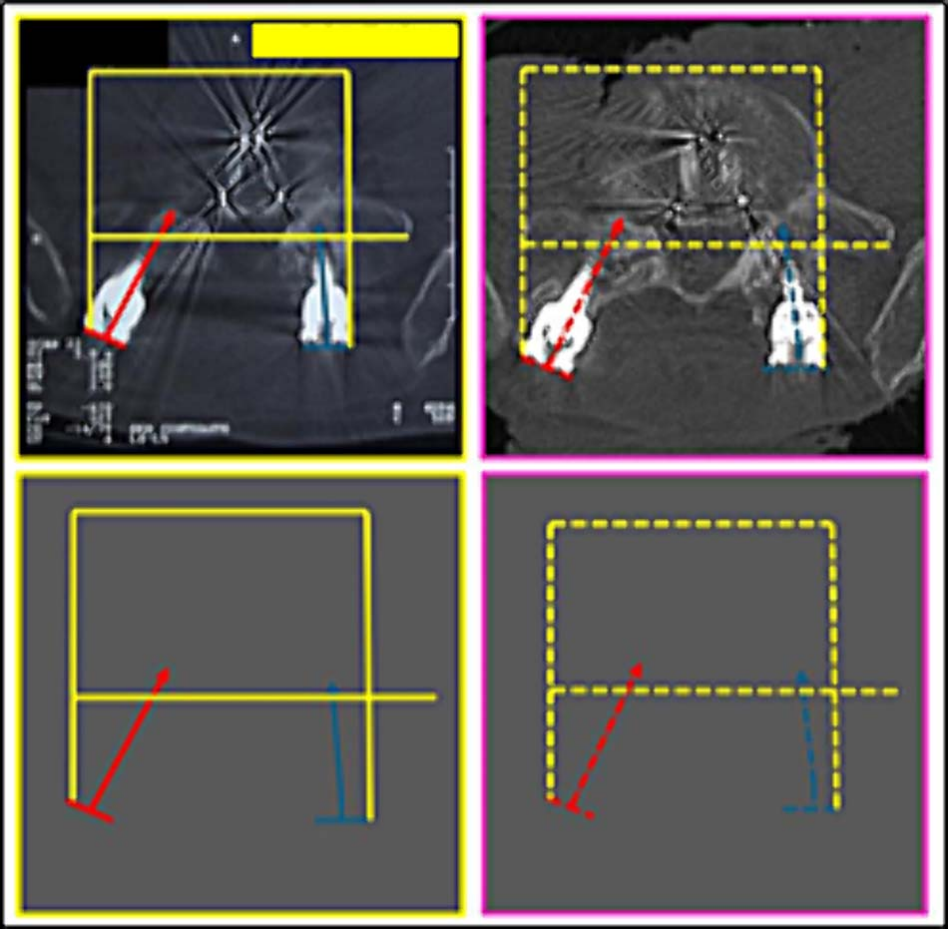
L5 vertebral body. Upper panels, comparison between the axial slice images of the antemortem (left) and postmortem (right) CT scans. Lower panels, respective matching templates.

**Figure 20:**
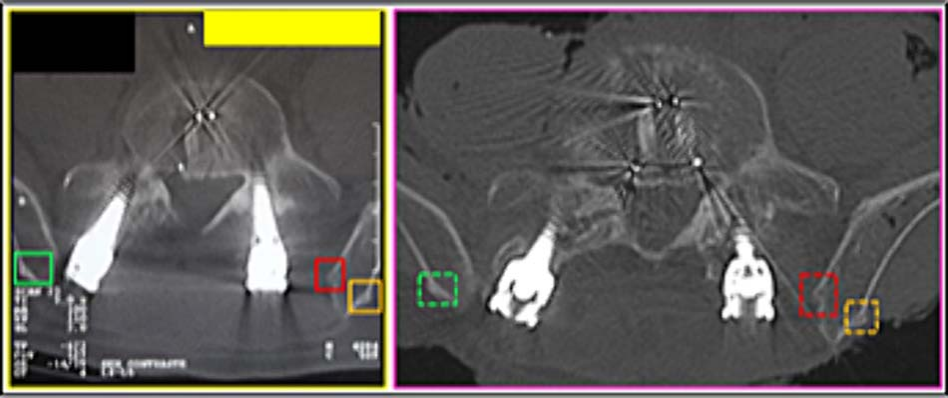
L5 vertebral body. Comparison between the axial slices of the antemortem (left) and postmortem (right) CT scans. Consistent elements.

(9) the relationship between the contour of the L3 vertebral body (lines), the stems (inferior circles), and the arrangement of the disc-replacement interbody devices (“cages”) (superior circles) (Figures 15 and 16),

(10) the morphology of the lower articular process of the L4 vertebra and the upper articular of the L5 vertebra (left superior square); the arrangement of the relationship between rod and bolt (left inferior square); the arrangement of the relationship between stem and joint processes (right square) (Figure 17),

(11) the contour of the L5 vertebral body (straight lines); the arrangement formed between the disc-replacement interbody devices (“cages”) (triangular shape diagram); the arrangement of the relationship between rods (circles) and screws (corner shape lines) and the posterior arch (Figure 18),

(12) the contours of the L5 vertebral body (straight lines); the interface of screws with the posterior arch structures; the angulation of the screws, individually and in groups (oblique arrows) (Figure 19),

(13) the bony relief morphology of the inner (superior right square) and outer (sideways squares) surfaces of the iliac crest (Figure 20),

**Figure 21:**
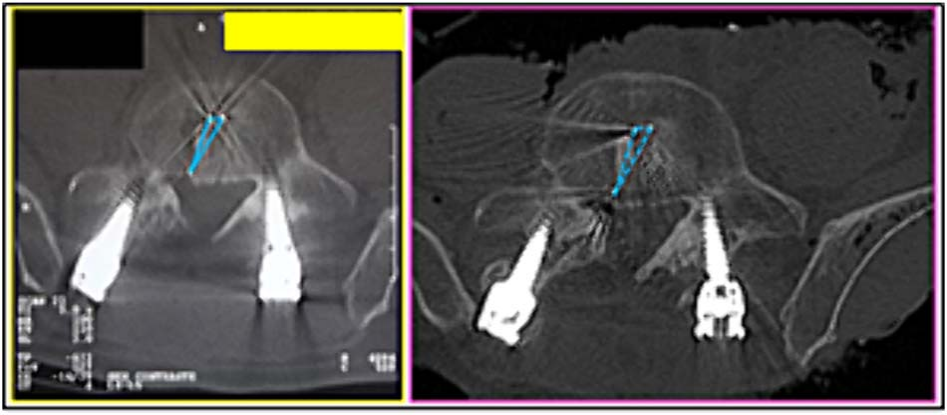
L5 vertebral body. Comparison between the axial slice images of the antemortem (left) and postmortem (right) CT scans. Consistent elements.

**Figure 22:**
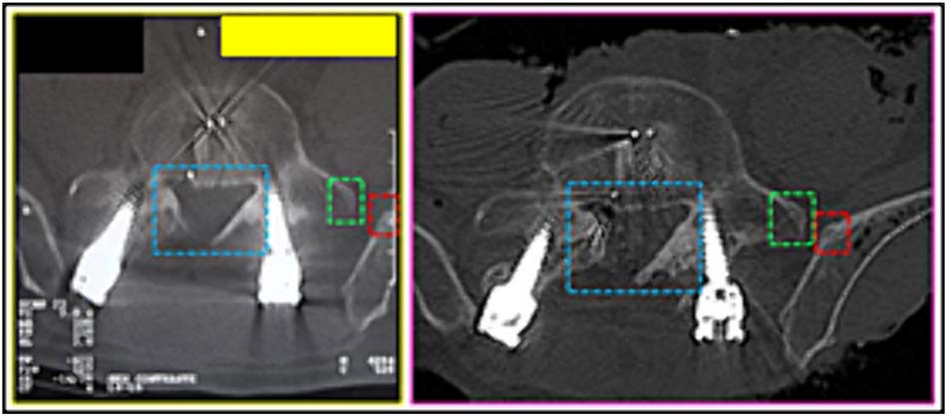
L5 vertebral body. Comparison between the axial slice images of the antemortem (left) and postmortem (right) CT scans. Consistent elements.

**Figure 23:**
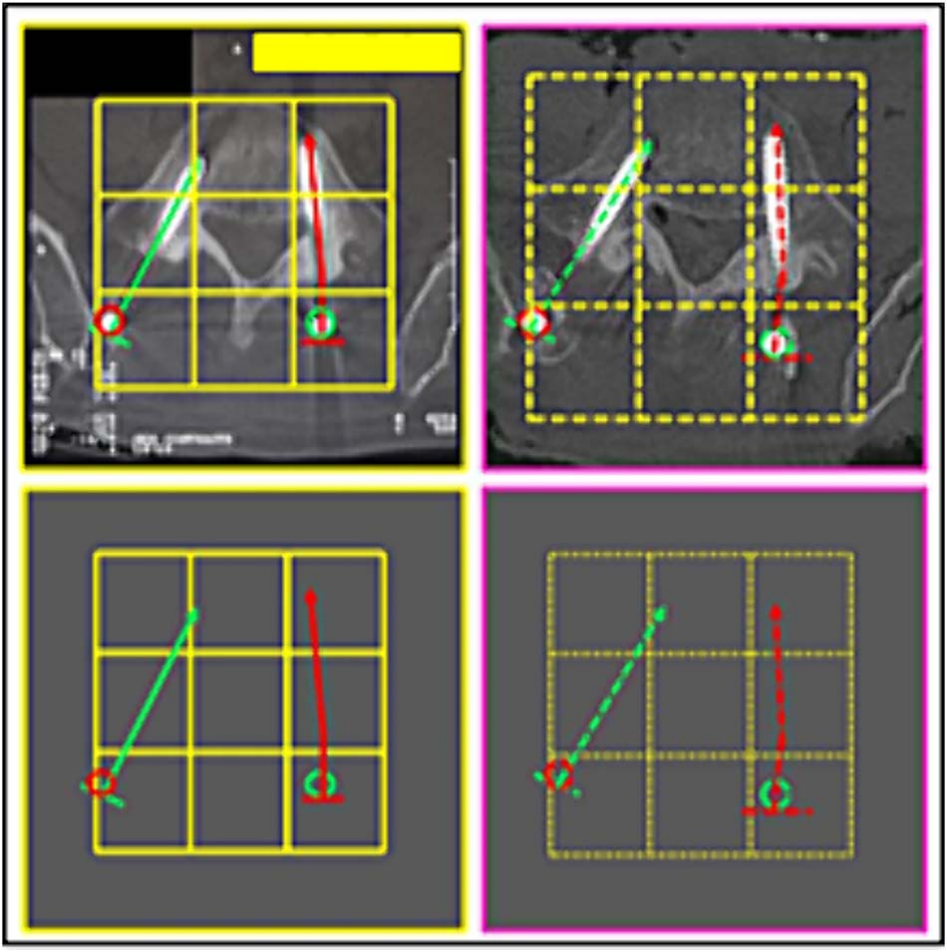
L5 vertebral body. Upper panels, comparison between the axial slices of the antemortem (left) and postmortem (right) CT scans. Lower panels, respective concordant templates.

**Figure 24:**
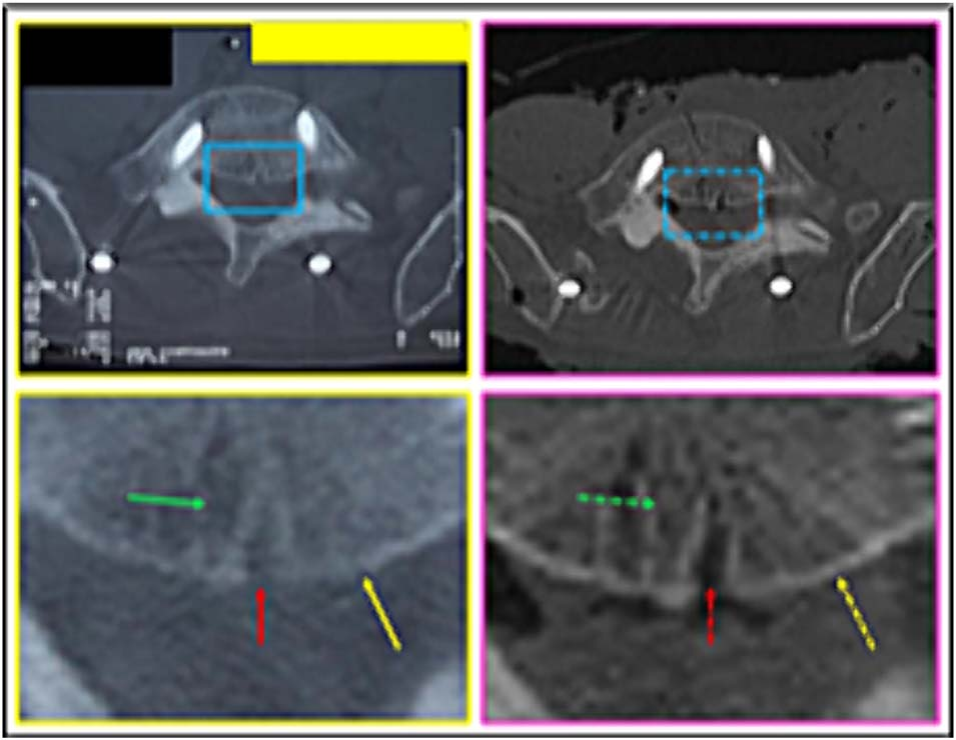
L5 vertebral body. Comparison between the axial slice images of the antemortem (left) and postmortem (right) CT scans. Consistent elements.

(14) the arrangement formed between the disc-replacement interbody devices (“cages”) (triangular shape diagram) in the fourth lumbar intervertebral space (Figure 21),

(15) the morphology of the L5 vertebral foramen (central square), the bone relief of its transverse process (right superior square), and the inner surface of the iliac crest (right inferior square) (Figure 22),

(16) the outline of the L5 vertebral body (straight lines); the relationship of the screws with the posterior arch; the angulation of the screws (oblique arrows), individually and in groups; the arrangement of the longitudinal bars (circles) (Figure 23),

(17) the indentation (central arrow) and bony trabeculate (sideways arrows) of the posterior contour of the L5 vertebral body (square) (Figure 24), and

**Figure 25:**
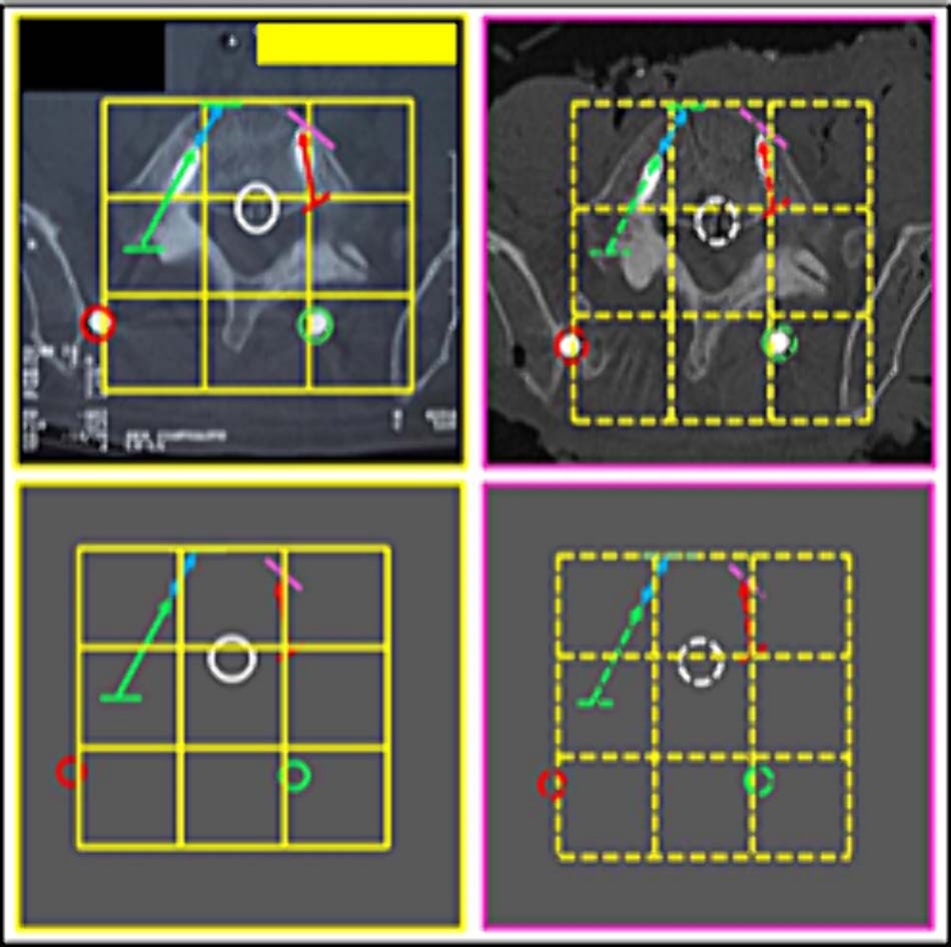
L5 vertebral body. Upper panels, comparison between the axial slice images of the antemortem (left) and postmortem (right) CT scans. Lower panels, respective concordant templates.

(18) the outline of the L5 vertebral body (straight lines); the relationship of the screws with the posterior arch; the angulation of the screws, individually and in groups (inferior oblique arrows); the relationship between the screws and the anterior contour of the vertebral body (left superior oblique arrow and right superior oblique bar); the indentation of the posterior contour of the vertebral body (superior circle); the arrangement of the stems (inferior circles) (Figure 25).

The context, consistency of the biological profile, presence of detailed matching points, and absence of excluding elements provided the technical and forensic elements necessary to establish a positive identification.

**Figure 26:**
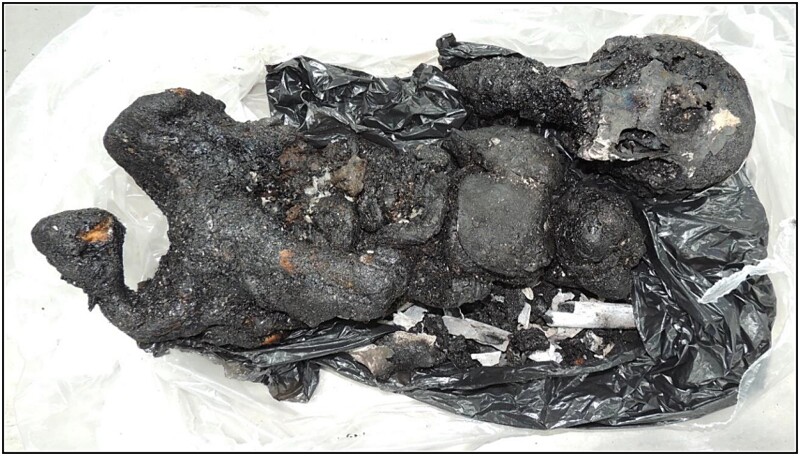
Remains of the charred body before examination.

**Figure 27:**
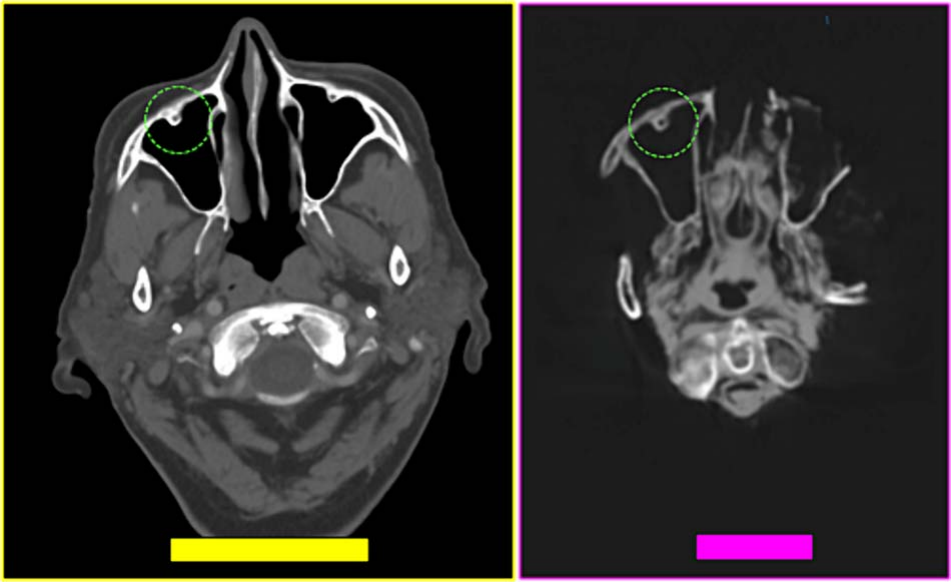
Coincident morphology of bone relief on the anterior wall of the right maxillary sinus (circumference) in the antemortem (left) and postmortem (right) CT scans.

### Case 3

After an accidental house fire, a charred body was found in a bed. The biological profile was assessed by the SAF/IMLAR team. The victim was confirmed to be female after her uterus was identified among the remains. Carbonization precluded anthropological method-based age interval estimation. Osteophytosis in the vertebral bodies and diffuse calcification of the entire aorta and iliac arteries visualized by CT scan suggested that the person was not young. Height estimation was also not possible from the absence/destruction of the long bones and metatarsals (Figure 26). A radiological examination of the body revealed identification factors, such as signs of neurological surgery, through a hyper dense image consistent with a vascular clip.

The investigators recovered the medical documentation of a missing person with a compatible biological profile, as well as a portable media device (“CD”) containing a CT scan of the skull, which provided further identification factors.

**Figure 28:**
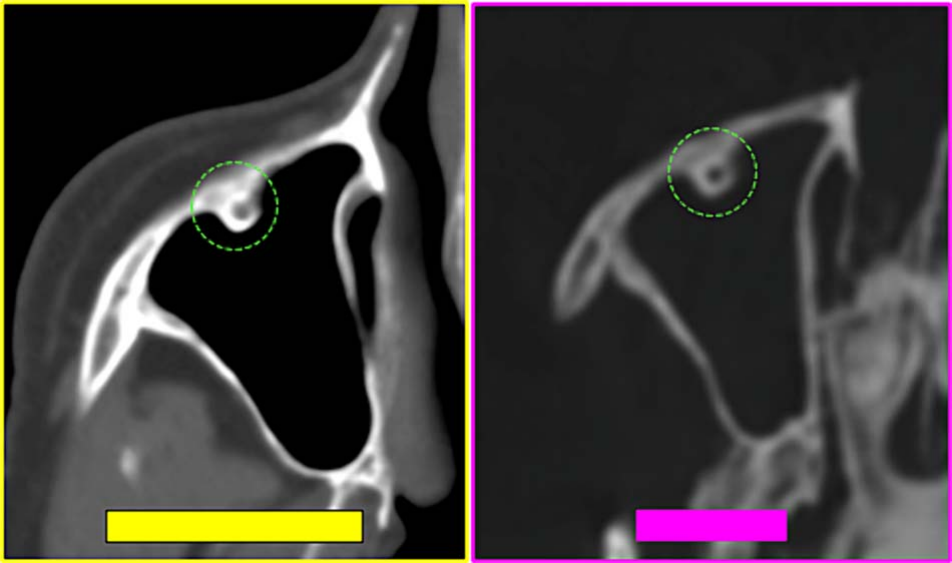
Coinciding morphology details of bone relief on the anterior wall of the right maxillary sinus (circumference) in the antemortem (left) and postmortem (right) CT scans.

**Figure 29:**
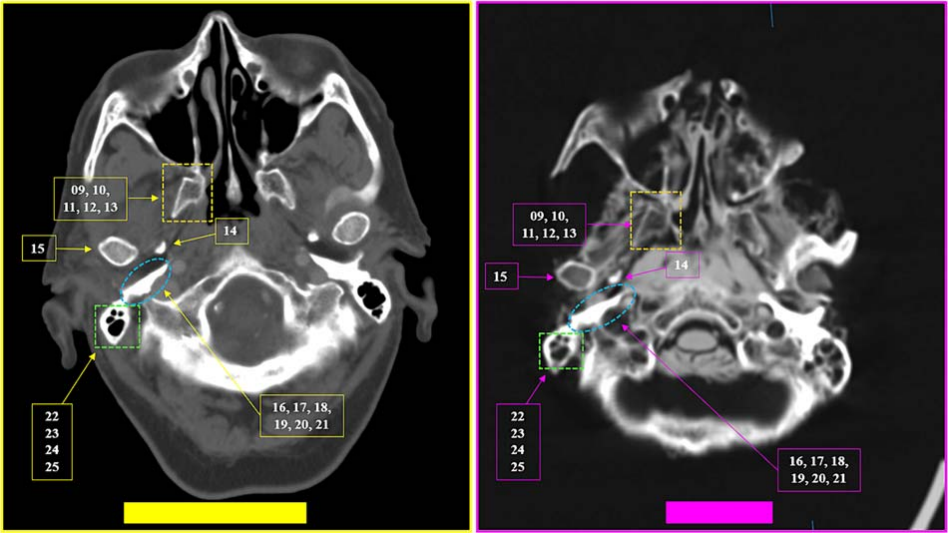
Multiple coinciding points in the cranial anatomical points and in the morphology arrangement of the right mastoid cells (left inferior square) in the antemortem (left) and postmortem (right) CT scans in axial sections.

**Figure 30:**
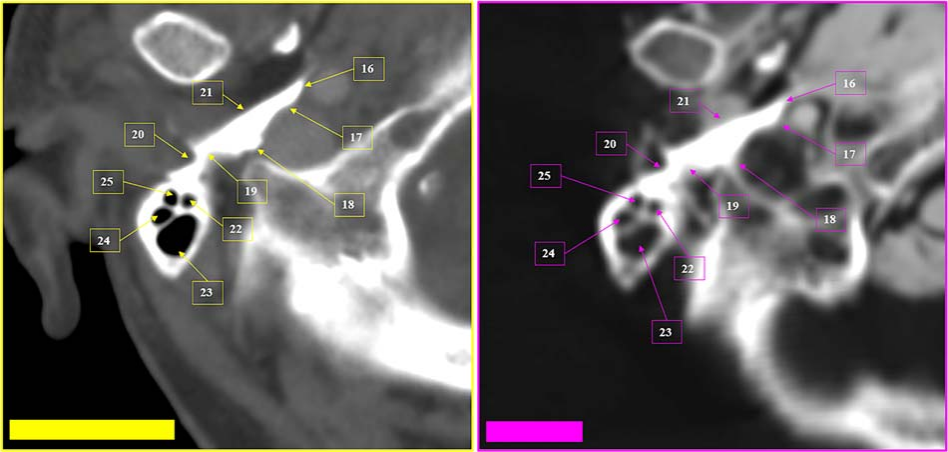
Coinciding morphology details and the arrangement of right mastoid cells in the antemortem (left) and postmortem (right) CT scans in axial sections.

**Figure 31:**
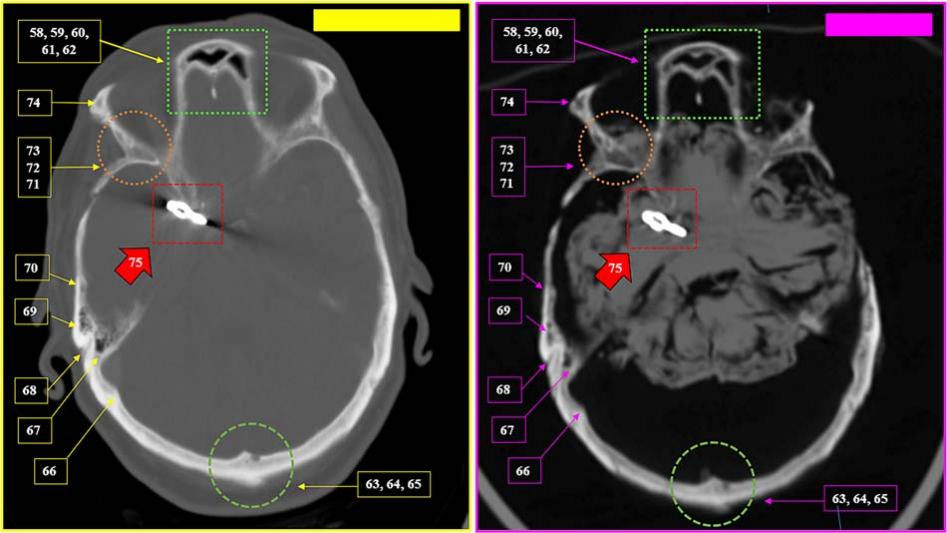
Matching morphology and arrangement of the frontal sinus ethmoid recess (superior square). Coinciding morphology and location of a device consistent with a vascular clip used in neurosurgical procedures (solid arrow). Coinciding morphology of bone relief on an internal table of the skull in the internal occipital crest (inferior circumference). Multiple coinciding points in the cranial anatomical points in the antemortem (left) and postmortem (right) CT scans in axial sections.

**Figure 32:**
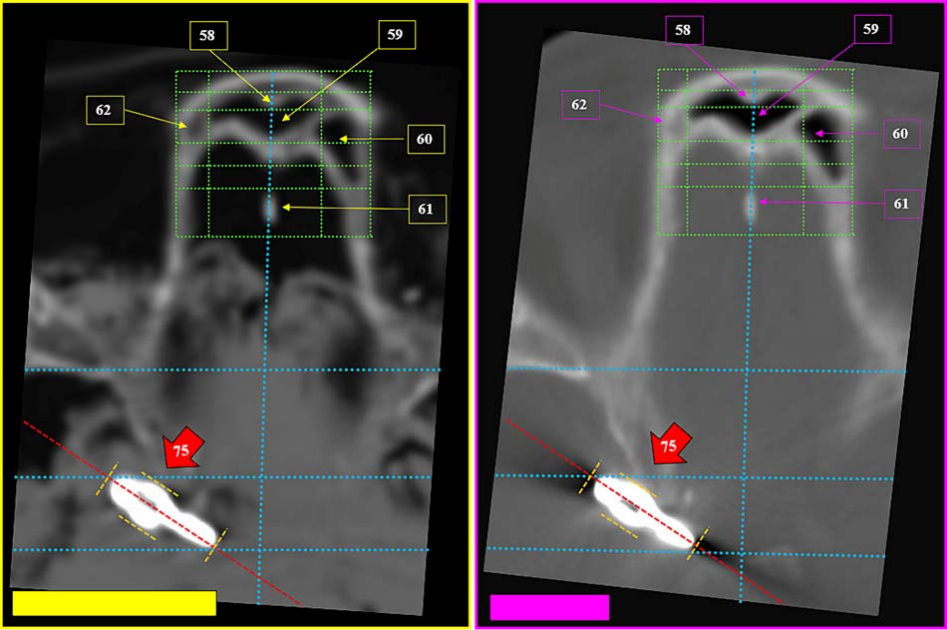
Details of the matching morphology and arrangement of the frontal sinus ethmoid recess (superior squares). Coinciding morphology and location of a device consistent with a vascular clip used in neurosurgical procedures (solid arrow). The oblique line represents the inclination of the clip in relation to the anatomical points (straight lines), highlighting the coinciding points between the antemortem (left) and postmortem (right) CT scans in axial sections.

**Figure 33:**
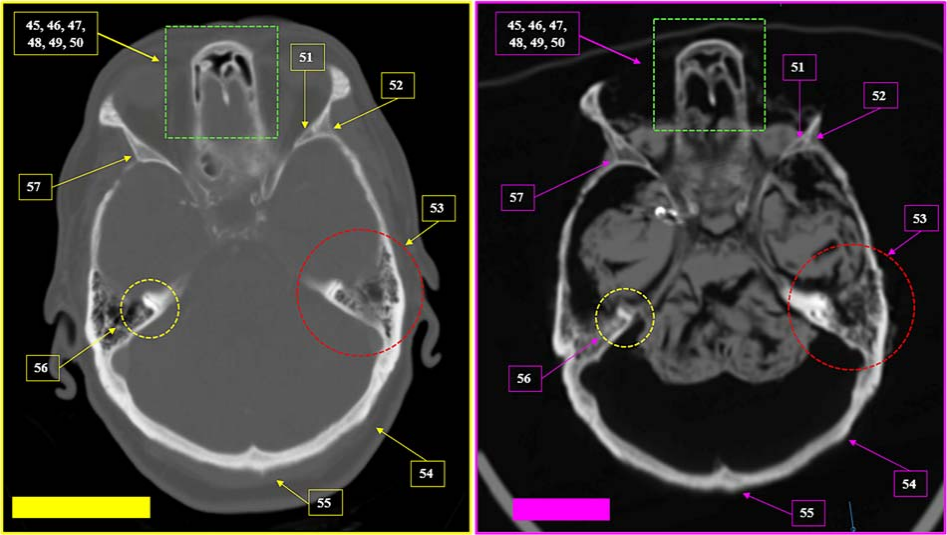
Matching morphology of the frontal sinus ethmoid recess (superior square); multiple coinciding points in the internal table of the skull in the antemortem (left) and postmortem (right) CT scans in axial sections.

**Figure 34:**
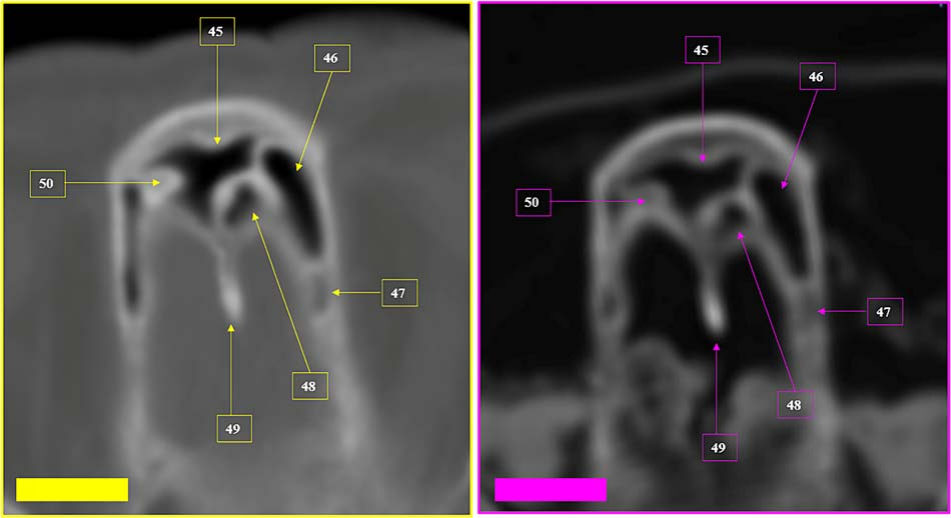
Details of the matching morphology of the frontal sinus ethmoid recess in the antemortem (left) and postmortem (right) CT scans.

A tomographic study was then performed on the remains. The anthropological and imaging comparative examination showed the following matching points (Figures 27–30), with no excluding elements:

(1) bone relief on the anterior wall of the right maxillary sinus (circumference) (Figures 27 and 28),
(2) multiple points of coincidence in the skull structure and morphology and arrangement of the right mastoid cells (left inferior square) (Figures 29 and 30),
(3) the morphology and arrangement of the frontal sinus ethmoid recess (superior square); the morphology and location of a device compatible with a vascular clip used in neurosurgical procedures (solid arrow); bone relief on an internal table of the skull in the occipital region (inferior circumference) (Figures 31 and 32), and
(4) the morphology and arrangement of the frontal sinus ethmoid recess (superior square); and multiple points of coincidence in the internal table of the skull (Figures 33 and 34).

The context, consistency of the biological profile, presence of detailed matching points, and absence of excluding elements provided the technical and forensic elements necessary to establish a positive identification.

## Discussion

The cases presented here clearly demonstrate the crucial roles of teamwork and, most importantly, interdisciplinary work between the pathologists, radiologists, and forensic anthropologists [10–12]. Identifying highly decomposed remains can be extremely challenging, and while some professionals are trained to assess macroscopic details in bones, others use imaging methods for further assessment. The meticulous work associated with comparatively analysing antemortem and postmortem images corroborates the importance of this strategy [13], which is a long and exhaustive task requiring complete focus. Several images from the cases were processed with Microsoft PowerPoint software to highlight the matching points, both in terms of shape and size (and proportion), as well as location. Therefore, these results could only be achieved through the close interactions and effective communication among the different experts involved in the identification process [12]. The technical details of the postmortem images are paramount for creating a reliable replica and performing an effective comparative analysis with the antemortem images. However, we want to emphasize that no editing or imaging adjustments were performed during this process. This is a crucial legal concern and fundamental for the success of the case. The collection/recovery of antemortem information is also extremely important and should be performed by a well-instructed forensic expert. In the cases described in this study, the recovery of antemortem documentation was carried out by the experts themselves.

The forensic anthropology community appreciates the need to develop a standard system for assessing concordant features to facilitate positive identification through radiographic comparison [10]. Ross and collaborators [13] attempted to develop a standard system in 2016, emphasizing that “the judge is the gatekeeper as to who qualifies as an expert witness and what scientific or technical evidence can be introduced in court”. Notably, no consensus has been reached regarding the minimum number of corresponding traits needed for identification, even for forensic odontology [13, 14]. In forensic anthropology, the method proposed by Ross et al. is currently the only one able to determine the minimum number of concordant features that characterize a high positive identification probability. This method can also provide the distinction threshold between correct and incorrect matches. The procedures followed in the cases presented here are clear and easily understandable by a court of law, as well as being corroborated by multiple matching points. Therefore, this method must be performed by a multidisciplinary team, as it is a meticulous and rigorous examination that requires a strong understanding of both anatomy and imaging techniques. Only experienced experts may identify some of the small and subtle anatomical details. In cases involving remains in an advanced state of decomposition, the forensic anthropologist should guide the experts and recommend which biological identifiers could be applied for identification. The extent of decomposition will dictate the method of choice, as fingerprints, odontology, and genetics-based approaches are often impossible to apply in late stages of decay. Therefore, assessing the main forensic anthropology parameters is fundamental for creating a profile and comparing it with a list of missing persons or possible suspects. This is the first step to effectively eliminate individuals, although the list of possible suspects may remain extensive. The second step is to investigate the individualizing characteristics, which are the numerous anatomical, pathological, or traumatic features that can potentially be used to differentiate a set of remains from others. Although rarer features have higher potential for identification, some frequently observed lesions, such as osteophytes, can also support identification because of their uniqueness. In the abovementioned cases, four main types of bone identifiers were exploited, including bone trabecula (absolutely unique), bone lesions, surgical devices, and skull sinus. These features, both individually and in combination, were critical for establishing a positive identification. The importance of forensic radiologists is also relevant as to guide postmortem examinations, replications, and comparisons with antemortem examinations [15–19]. Additionally, effectively obtaining antemortem records is fundamental for a successful identification.

From a forensic anthropology perspective, identifying unknown human skeletal remains poses several methodological challenges from various factors. Additional identification factors can contribute to the overall forensic reconstruction of an individual’s life history, fostering the potential for individualization. The cases described in this study provide evidence that positive identification remains a possibility, even with extensively decomposed remains. Forensic anthropology should certainly not be considered as a last resort in identification. Instead, forensic anthropologists should participate in cases involving decomposed remains from the very beginning to help monitor the procedures.

## Conclusion

Choosing the most efficient method for identification in a forensic case is dictated by how preserved the human remains are. Forensic anthropologists, as well as other forensic stakeholders, should be familiar with a broad spectrum of methods, including their scope and limitations, to be able to perform or recommend all approaches necessary for a successful identification. All identifiers should be designated as biological identifiers rather than polarizing primary *versus* secondary identifiers [12]. Overall, our findings corroborate the position of the Forensic Anthropology Society of Europe (FASE) that forensic anthropology identifiers are valuable tools for biological identification [20, 21]. Despite the advanced state of decomposition of the remains described in these three cases, unique anatomical, pathological, and surgical findings contributed to successful identifications. This was achieved through meticulous radiological comparisons using the shadow technique, as well as both digital radiography and CT images. Technological advancements have greatly improved the chances of identification of putrefied and charred remains, further ensuring that the possibility of identification should never be ignored.
